# An Essay on Odontalgia, Its Causes and Its Treatment

**Published:** 1845-03

**Authors:** S. E. Arms


### ARTICLE V.

*An Essay on Odontalgia, its Causes and its Treatment.* Read before the Medical Society of the county of Essex, N. J., in May, 1844. By S. E. Arms, M. D.

It would have been more gratifying to my feelings, had the members of the Medical Society of this county, selected some other individual to fill the office of president, and to address you at this anniversary meeting. But as you were pleased, at our last annual meeting, to re-elect me to that office, I shall not shrink from an humble effort to discharge its duties, in addressing you on this occasion.

The subject which I have selected as the theme of a few observations, is odontalgia; its causes and its treatment. Among the various ills to which flesh is heir, few, perhaps, occur more frequently, none, in its acute form, is more trying to bear with patience; and none, probably, excites less sympathy than that of tooth-ache. The symptoms which characterise this affection, in ordinary cases, are too well known to need a description. Few who have arrived at adult age, have been so fortunate as not to have had painful experience of this disease.

The pathological condition of teeth that are painful, is not, perhaps, as well understood as it should be. Most medical and dental writers, however, agree that, in the acute form of the disease, its proximate cause is an inflammation of the internal periosteum, or lining membrane of the tooth. To avoid a misunderstanding, I will here observe, that whenever I use the term lining membrane, in this essay, I mean that exceedingly delicate structure, which passes up from the extremity of each fang, to the internal cavity in the central part of each tooth; surrounds the nerve and blood-vessels, in each of their delicate canals, and also that nervous, pulpy and vascular mass, which occupies this cavity.

The membrane which surrounds each fang in its alveolus, might, with propriety, be called the peri-dental membrane, and the reflected portion of it, which lines the alveolus, might be called the peri-alveolar membrane. But I much prefer the term *investing* membrane, to designate that part which covers each root in its socket, and *alveolar* membrane, to designate that which lines the socket of each root. Much obscurity and misapprehension often arise on medical, as well as other subjects, because terms are not accurately defined. Every writer is privileged to use terms in the way he pleases, provided he defines them accurately; yet good taste and good usage should be our guide in these matters.

That the lining membrane is in a state of inflammation, and that of an active kind, in a majority of cases of acute odontalgia, there is not a doubt. Though the pain is doubtless much more acute, than in any other part of the system subject to the same degree of inflammation; and for obvious reasons. The lining membrane is highly vascular, and surrounded externally with osseous walls, as unyielding as marble, while it encloses internally, a structure which in delicacy of organization, and sensitiveness upon the approach of external irritants, exceeds, probably, that of every other structure in the body. If the ordinary characters of local inflammation are present in this membrane, there must be an increased flow of arterial blood to the part, and consequently a tumefaction or thickening of its parieties. The cause of pain, under these circumstances, is very obvious. As the walls by which it is surrounded externally, cannot yield, the nervous pulp is compressed on every side, and more or less violently, according to the extent of the inflammation. In addition to this, the nervous pulp is supplied with blood-vessels within its own substance, which I have often noticed, much engorged with arterial blood. In what is vulgarly called jumping tooth-ache, there is apparently a violent augmentation of pain at every pulsation of the arteries.

In speaking of the remote causes of odontalgia, there is a wide field open for investigation; for they are as various as the causes of caries in the teeth.

There are, doubtless, cases where the teeth become painful, while these organs remain quite sound; but these cases occur so rarely, that we may properly consider them as exceptions to the general rule, that they become painful in consequence of decay. Hence an inquiry into the causes of decay in the teeth, which is unfortunately so common at the present day, is by no means destitute of interest.

I shall not attempt in this essay, to give the views of the different authors who have written upon this subject.

Among the most celebrated in the last century, is John Hunter, whose work was published in London, in 1778. He describes, with clearness and precision, the result of investigations previously made, and also those made by himself. It is well known that he was an advocate of the non-vascularity of the teeth. His opinion was based mainly on the result of his experiments of feeding young animals upon madder. He found that those layers of the teeth only were reddened, that were formed while the animal was feeding upon the madder; but the accuracy of his experiments has been doubted, and the vascularity of the teeth has been incontestibly proved. According to Fox, whose celebrated work appeared in 1803, the cause of decay in the teeth, is inflammation terminating in mortification.

The next important work on the teeth, that appeared in Great Britain, was that of Thomas Bell, in 1829. He holds that the teeth possess vitality, and are connected, by their organization, with the general system, having nerves, blood-vessels, and absorbents, and are analagous, in this respect, to true bone. He was a strenuous advocate for a degree of vitality in the teeth equal to bone, and adduces as proof, the red tinge in the teeth of persons who have died of strangulation. The fact that the teeth of those who have died of certain varieties of asphyxia, particularly from drowning, are almost uniformly tinged with red, was noticed many years since by the late Professor Hayden, of the Dental College, Baltimore, recently deceased. He found, that if examined immediately after death, they presented a deep pink hue, if some time after, the tint was darker. The different shades of color depend also, in a great measure, upon the age of the person and the violence of his death. The late Edward Hudson, formerly a distinguished dental practitioner of Philadelphia, has, in several instances, noticed the same phenomenon.

When we review the writings of the different authors on the minute anatomy of the teeth, during the last two centuries, we find their views are, on many points, quite contradictory. I shall not attempt to discuss the merits of the different views which have been entertained by writers of the English, French and German schools, on the formation and uses of the lining, investing, and alveolar membranes, and on the minute structure and functions of the internal parts of the teeth; but I cannot pass over in silence, the original and ingenious experiments of Professor Retzius, of Stockholm, recently published. He procured, with the aid of a file, slices of tooth-bone as thin as writing paper; increased their transparency by means of olive oil and turpentine varnish; and on examining them under a powerful microscope, found they were composed not merely of hollow fibres, but of ramifying tubes, of which the trunks open into the cavity of the tooth, supporting themselves upon its pulp, while their branches run towards the external surface. In fully developed teeth, these tubes, for five-sixths of their course from their commencement, in the internal cavity, appear to be of the same diameter, and this diameter, Retzius, with the aid of the micrometer, has ascertained to be $\frac{1}{385}$ part of a line. In their remaining course, their diameter diminishes considerably, until they disappear, or terminate in small irregularly circular scattered cells. When very thin and transparent slices were examined in a clear day, under a glass of 300,500 times magnifying power, it is found that these canals are main tubes with dichotomous branches, and giving off, in their entire course, an infinite number of small subdividing branches. The experiments of the learned professor were conducted on a very extensive scale, not only upon human teeth, but upon those of a large number of animals of the class mammalia, amphibia, and fishes; and while peculiarities were noticed in the specimens from different animals, all were found to agree in having essentially the same tubular structure. The horns of the deer, though organized in nearly the same way, were found to contain real blood-vessels. With regard to the uses of these tubes, our author speaks with a becoming candor. He allows he is ignorant of their functions, but gives it as his opinion that they convey a nourishing fluid. This, he supposes, does not continue through life, but that in later years, the process is carried on without a constant exchange of solid matter. Mr. Nasmyth, of London, has been instituting a series of experiments, commencing where those of Retzius terminated, and the results of his experiments, which are looked for with interest, it is expected, will soon be given to the public.

If there be a circulation in the dental bone, as I fully believe there is, and as I think I have had demonstrative evidence to prove, it doubtless becomes more and more feeble as a person advances in age, and finally ceases altogether; as it is well known that the dental canal becomes quite obliterated in aged people. Subsequent to this, it is absurd to suppose that any circulation exists. The same, I think, is applicable to the nerves. That the whole tooth, the enamel possibly excepted, is furnished with minute nerves, is demonstrated in the daily observation of every dental operator. This is certainly the case, unless there can be a high degree of sensibility without the presence of nerves. A fact I have noticed in a multitude of cases, of a much higher degree of sensibility in that part of a carious tooth immediately beneath the enamel, would seem to show the existence of nervous filaments in that part, to a greater extent than in other parts of the dental bone.

The late Professor Hayden, of Baltimore, claims to have discovered a membrane immediately beneath the enamel, which lines it internally, and forms a bond of union between the enamel and bone. The same membrane is described by Retzius, though the discoveries of each were doubtless made without the knowledge of the other. My observation has led me to conclude that there is a higher degree of organization and sensibility in this membrane than in the bone beneath. But fortunately, we need not depend upon the opinions of writers, who, while we acknowledge their erudition, are often contradictory in their statements, to prove or disprove the vascularity of the teeth. That they are vascular, has been demonstrated in two instances, by Professor Harris, of Baltimore, who has given a beautiful drawing and description of an inferior molar tooth, taken from a boy eleven years of age. The tooth had ached violently, for several days, and on splitting it open, not only were the vessels of the pulp injected with red blood, but with the aid of a microscope, a number of vessels within the very substance of the bone, were discovered charged with this fluid. This drawing may be found in the American Journal and Library of Dental Science, for March, 1842. Also in the same Journal, for September, 1841, may be found a similar drawing, from Charles Brown, of Woolwich, England. I will add, that on the 29th of March last, 1843, I extracted a superior molaris, for a young man about 20 years of age, and on splitting open the tooth, found evidence of vascularity in the dental bone, almost as decided as in the cases alluded to. The tooth had ached violently at times for the last eight months.

I have adduced these facts, because I consider a correct anatomical knowledge the only sure guide to sound practice. It has been suggested, that after filling teeth, the vessels that are cut off by excavation deposit bone beneath the filling. Possibly this may be correct, but I can recollect no facts which go to prove it. But to return to the remote causes of decay, and consequently of pain in the teeth. My opinion is, that it is chiefly caused by external agents, and consequently, that it is more directly under the control of individuals, than is generally supposed. To this opinion, I have been recently led, by facts that have come to my knowledge, and a careful review of my own experience. That there is a predisposition to decay, in the teeth of some, as there is a predisposition to phthisis in others, I freely admit; but even in these cases, much may be done to retard the action of external corrosive agents upon these useful organs. Many cases occur, where a tooth has a fair exterior, and is, to a superficial observer, entirely sound, but upon a critical examination, after penetrating the enamel, a large portion of the tooth will be found to be destroyed by caries. Cases of this description, have been adduced as arguments to prove, that the causes of decay are internal. They were so regarded formerly, by myself. But upon calling my attention more particularly to the subject, and recalling former experience, I am led to believe, that a case can scarcely be produced, where there is caries in the bony part of the tooth, without an aperture in the enamel sufficiently large to admit the air, and fluids of the mouth; and it is well known that these fluids are often exceedingly acrid, and will act with great rapidity upon the osseous structure, when this safeguard of the tooth is removed.

If these views are correct, it follows that a perfect enamel over every part of the crown of the tooth, is of the first importance to preserve these organs in a state of integrity. It becomes us, then, to inquire into the causes of defect in the enamel. Is this owing to the same causes as decay in the osseous structure? usually I think not. It is owing, in many cases doubtless, to imperfect organization at some particular points; and to this, nothing, probably, contributes more than a crowded condition of these organs.

It is well known that the incisors, cuspidati, and bicuspids, decay far more frequently upon their lateral surfaces, than elsewhere. This is just what might be expected where they are in such close contact that they have not an opportunity fully to develope themselves. My views on this subject may be familiarly illustrated by referring to two or more young trees that are in close contact while they are in a growing state. It is manifest that not only the cortical, but the ligneous fibres of the tree would be greatly injured. In illustration of the correctness of these views, I have often witnessed cases where every tooth that was in close contact with its neighbor was decayed upon its lateral surface, while those which had a natural separation between them, were entirely sound.

In the molares, the decay oftener commences in the depressions in the crowns. At these points, and about the necks of the teeth, the enamel is always much the thinnest; so that a little imperfection in the organization, or mechanical violence, as that of cracking nuts, biting threads, or masticating any substance unusually hard, or the sudden alternations from heat to cold, and vice versa, when the drinks and food are taken into the mouth, might cause a chink or seam at these points. And let it be borne in mind, that the air and fluids of the mouth are penetrating almost beyond our conception, and might find access to the bony surface through an aperture that was not perceptible to the naked eye. These depressions, and the interstices between these organs, afford, moreover, a convenient place for particles of food and other acrid substances to remain and undergo a fermentation, which would render them exceedingly corrosive.

The too early extraction of the infant teeth, may also conduce to the imperfection of the enamel of the permanent ones. Another view of this subject, affords, I think, a most conclusive argument in favor of the position I am advocating. If the causes of caries are internal and constitutional, I can see no reason why the decay should not fall upon the fangs of the teeth, as often as upon the crowns. That this is the case, is not, I believe, contended by any.

In this connection,* I cannot forbear mentioning the case of a gentleman, a captain in our navy, that came under my notice in the fall of '43. He having suffered from hemoptisis and an enfeebled condition of the system, had been using the elixir vitriol, by the advice of his physician, for two or three months. He received no specific directions from his physician, as to the *manner* of using it; and without any suspicion of mischief arising therefrom, had taken it daily, without any precautions whatever. The result in this case, as might have been anticipated, was truly lamentable. Almost every tooth he had, was more or less corroded about its neck; but the inferior incisors were so decayed at these points, extending deeply beneath the gums, that it was with great difficulty they could be preserved by filling. In his case, the enamel in its thickest parts had

* The author alludes to certain experiments, which he describes, as having instituted with a view to test the action of particular chemical agents on the teeth, but which are here omitted.—Eds.

served as a protection from the acid, but on the lateral surfaces of the inferior incisors, next the gums, where they were almost or quite destitute of enamel, and where, by its gravity, the acid would be likely to fall, it was left to act with all its force.* Tartar or salivary calculus, is another cause of decay, though the ordinary yellowish kind, has a more direct tendency to cause a chronic inflammation in the gums, extending to the alveolar and investing membranes, which causes a loosening and final evolution of the teeth from their sockets, than to cause decay. There is a dark greenish kind, however, which appears to possess highly corrosive qualities, and soon causes the teeth to decay.

In many cases, there appears to be a predominence of acid in the fluids of the mouth, which exerts a very deleterious influence upon the teeth. And as the state of these fluids, depends upon the state of the general system, as well as upon the food, drinks, condiments, and medicines, taken into the mouth, the teeth may be said to decay indirectly from constitutional causes. A morbid condition of the system, while the teeth are in a forming state, has often a great influence in preventing their healthy and perfect developement.

There are, perhaps, no cases that come under the notice of the physician, where sympathetic action is more manifest than in some cases originating in morbid irritation about the teeth and jaws. Cases of acute pains in one or several teeth, extending to the gums, the muscles of the face, jaws and throat, and often to the ear, are frequently met with. The patient is usually exceedingly sensitive upon the approach of cold air, or cold drinks, and can, with difficulty, open the mouth. Of the severity of the suffering in cases of this description, I can testify, from painful experience. It was, I think, in the spring of 1835, that I had an attack of this nature. The pain was of the most excruciating character. A variety of applications had been

*It is incumbent on the physician to give specific directions, as to the manner of taking all medicines that can possibly injure the teeth—and also to urge upon the patients, the importance of unusual attention to cleanliness of the mouth and teeth, during sickness, as the vitiated secretions will often act upon the teeth with great energy at this time.

made without a perceptible diminution of the pain; had slept little for one or two nights. The pain continued to increase, until it finally arrived at such a pitch, that I could only walk the house in agony.

While in this predicament, the thought occurred to me that the conium maculatum (called, improperly, in the Shaker's catalogue, cicuta leaves) might, perhaps, prove beneficial, applied in the form of steam or vapor. I took about ʒ vi. of the dried leaves, threw them into a basin, and poured upon them boiling water. After standing a few minutes, the vessel being covered, I removed my cravat, freely exposed my face and neck, and setting the basin at a convenient height, threw over my head and shoulders a thick shawl that was at hand, so as to inclose the basin over which I leaned. I not only exposed my face and neck, but freely inhaled the vapor, which was made to rise, by throwing into the basin, at intervals, pieces of anthracite coal in a state of ignition. I had often heard professor Ives, of the Yale Medical College, while a pupil in his office, speak of medicines acting like a charm, but never until this, realized the meaning of the expression. So signal and almost instantaneous was the relief, that in less than five minutes I fell into a refreshing sleep. I have frequently been called upon by patients suffering in a similar manner, and have directed the conium, as described above, and with uniform success. And though the relief is not in all cases as speedy and complete as it was in my own, I have uniformly found that there was a material mitigation of the symptoms. It is necessary, however, to caution the patient against subsequent exposure. I have found an anodyne poultice, composed of onions, boiled very soft and blended with the petals of the papaver somniferum, a remarkably soothing application, applied immediately after the narcotic vapor is discontinued. In cases where the inflammation runs high, local depletion, by means of leeches, together with cooling purgatives, would, doubtless, prove serviceable: but I have usually resorted to the narcotic vapor at once, and found it eligible and highly effectual.

I have often been requested to extract one or more teeth for those who were suffering in this way, but have uniformly declined, as I am satisfied of the impropriety of the practice.

Many valuable teeth might be sacrificed without obtaining the desired relief. Furthermore, there is frequently, in these cases, such extreme difficulty in opening the mouth, that humanity would forbid making the effort, except as a dernier resort. The nerves of the face and jaws are in that peculiarly sensitive state, that a breath of air, or the slightest touch, is sufficient to add to one's agony. Nothing, under these circumstances, within my knowledge, is so soothing as the narcotic vapor.

Well authenticated cases of *mania* and phrenitis are on record, which originated from decayed teeth, and were relieved by their extraction. Also, severe rheumatic and auricular pains, which were excited by the same cause, and relieved by the same means. Cases of the latter, I have often witnessed and could relate in detail, did time permit.

When we glance, for a moment, at the anatomical structure of the jaws, face and head, the explanation of these phenomena will be obvious. We find the fifth and seventh pairs of nerves, which may be properly styled the maxillary and auditory nerves, originating in or near the pons varolii, near the basis of the brain, and forming a communication between the two, and, consequently, between the lower jaw and the ear, through the medium of the corda tympani, a small nervous cord, which passes downward, and forward, from the upper and posterior part of the cavity of the tympanum, through the fissure of the cavity for the head of the lower jaw, unites with the lingual branch of the inferior maxillary nerve, thus forming a direct communication between the lower jaw and the parts about the ear. The portio dura of the seventh pair of nerves, ramifies, extensively, upon the superior and inferior maxillary, malar and temporal bones, upon the temporalis, masseter, and orbicularis muscles, also upon the muscles of the cheek, nose and lips, and, finally, communicates with small branches of the *superior* maxillary nerve. It will thus be seen, that a nervous communication exists between the brain, the face, the ears, and parts contiguous.

*Treatment.*—We come now to speak of the treatment of odontalgia. As caries extending so deeply into the tooth, as to expose the dental canal, may properly be regarded as the usual cause of this affection, it will be proper, first, to say something of the means of arresting the ravages of this common malady, and, consequently, of preventing the tooth-ache.

It is my firm conviction, from years of observation, that the foundation for the ruin of the teeth is usually laid in childhood, and, mainly, by personal neglect. Comparatively, few ever think of troubling their teeth, until they trouble them. A sound and healthy constitution is, undoubtedly, necessary to insure, permanently, a sound set of teeth: hence, all those means which serve to develope a sound physical frame, will be necessary from childhood to adult age. In particular, should a frequent and faithful use of cold water be made to the gums of the infant, with friction, by means of the brush, or better, perhaps, in early infancy, the nurse's finger. As soon as the teeth pierce the gums, the nurse should see that this practice is persevered in, and that the mouth of the infant is effectually cleansed several times in a day. As the child attains sufficient age, it should be taught to use the brush, and by no means should its faithful use be intermitted for a day. Cold water should be freely used, but ice-water, or that which is extremely cold is objectionable. If this habit is early formed, it will prove not only agreeable, but exceedingly useful. If the child can keep the teeth entirely clean without the aid of a dentifrice, it should not be employed; but if he fails in this, a powder that possesses moderate mechanical pro perties, and is entirely free from acid of all kinds, should be immediately resorted to. Much judgment and circumspection are often necessary in the treatment, during the moulting of the infant teeth, and the eruption of the permanent ones. Not only should the infant teeth be extracted, when necessary, at the right time, but in some instances it is absolutely necessary in order to preserve the symmetry of the jaws and face, to extract one or more of the permanent teeth. It should be borne in mind, however, that as the child advances in age, a material expansion of the jaws takes place. This should prevent hasty and injudicious extraction.

Where extraction is necessary, the bicuspids are the teeth that should be selected for this purpose, and usually the second should be preferred to the first. The incisors should never be extracted, unless the case is very peculiar. If the extraction of the appropriate teeth, together with pressure in the proper direction, upon the deviated ones, is not sufficient to reduce them to a symmetrical form, a mechanical fixture should be applied to effect that object. But the detail of the treatment in these cases, would be inappropriate in this essay.

By adopting this course, two objects will be gained. The beautiful symmetrical arch of the jaws will be preserved, and the teeth being relieved from strong lateral pressure, will be far less liable to decay. Should they, notwithstanding, give evidence of decay, no time should be lost in having every particle of the caries eradicated, and if the cavity penetrates the tooth, it should be so effectually stopped with gold, as to utterly exclude all foreign agents from contact with the osseous structure of the tooth.

*Tartar* should be effectually cleansed from every part of the dental organs.

We have now to speak of *teeth with exposed nerves.*—Teeth in this condition are not always painful. If the exposure is but partial, and the lining membrane is not wounded, by great care on the part of the operator, the caries may be removed, and the cavity filled with gold. A very nice tact is, however, necessary in these cases, to thoroughly remove the decay and condense the filling, and at the same time, introduce it in such a way as not to press upon the nerve.

In this connection, I cannot avoid speaking, in the strongest terms of reprobation, of the practice of filling valuable teeth with an amalgam of mercury and silver. This composition has been lauded by the Crawcours and Mallans, of New York, and many other small fry who have followed in their footsteps, under the names of royal succedaneum, metallic composition, chinese cement, lithodeon, and a host of other names, but the composition is always essentially the same, viz. mercury and silver, or perhaps, a little tin, lead, or bismuth. Multitudes have been gulled, by its advocates, to their hearts' content. It is wonderfully convenient for those who have not the requisite skill to fill a tooth with gold. They may be appropriately termed tooth-masons, as it requires no more skill, than it does to plaster up a crevice with mortar or putty. It becomes exceedingly hard, it is true, and is sure to remain in the cavity, and is, consequently, the more mischievous. To say nothing of the salivation, and ulceration of the maxillary bone, extending even to the antrum, of which we have well authenticated cases; as it always contracts on hardening, in accordance with a well known chemical law, it affords an ingress to the fluids of the mouth, and moreover, the material itself corrodes with rapidity. Although apparently successful in some cases, the caries is always found to extend beneath it, as I can testify, from having witnessed many deplorable cases of this description. Many beautiful teeth that have come under my notice have been utterly ruined by it.

Every physician, and every intelligent member of the community, therefore, should reprobate the practice, in the strongest terms.

But to return from this digression. Teeth with exposed nerves, if left to themselves, are liable to become painful from the slightest causes. The least pressure or irritation is liable to excite inflammation, which may terminate in exquisite and continuous pain. In such cases, extraction is sometimes advisable, though not always. As a palliative, I have used, for many years, odontalgic drops, prepared according to the following recipe, with the most decided success:

℞ Ol Peppermint, ʒ iii.

Ol Cloves, ℥ ss.

Ol Cinnamon, ʒ iii.

Nitras Argenti, pure, ʒ i.

Tr. Opii, prepared with pure alcohol, ℥ iv. M.

I have found this more effectual than kreosote, and altogether more pleasant in the mouth. These cases were formerly considered as hopeless; though sometimes cauterized with a hot iron, or an alkali, or strong acid, and afterwards filled, the practice was seldom successful.

Dr. Spooner, of New York, some three or four years since, proposed the arsenious acid as an agent for destroying the nerve of a tooth, where it could not be prepared for filling without this dernier resort. The success of the application has far exceeded any thing that has ever been used for this purpose. It is astonishing to observe, how effectual so small a quantity as the twentieth of a grain will have when thus applied. My usual practice is, to combine the arsenic with about an equal part of sulphate of morphine. After removing so much of the caries as is necessary to expose the cavity containing the nerve, I saturate an exceedingly small piece of cotton with the drops above mentioned; this is retained upon the point of a small pointed instrument, and while moistened with this liquid, is introduced into the vial containing the morphine and arsenic, and touched lightly upon the powder, so as to cause a portion of it to adhere, of a less size than that of a small pin's head. This is then carried down so as to rest directly upon the exposed nerve, and is retained there by another piece of cotton, saturated in gum mastic, dissolved in ether. I direct the patient to remove it at the expiration of three days. As there is, usually, some soreness of the periosteum, and a sanious discharge from the cavity, for a few days, I usually wait from three to six days after removing the preparation, before proceeding to prepare and fill the cavity. At the expiration of that time, it can usually be accomplished with little or no pain to the patient. I think I have usually had best success where not only the carious part, but the debris of the nerve and blood vessels, occupying the dental canal, were thoroughly eradicated; sometimes, almost or quite to the extremity of the root, before stopping the cavity. In this manner, I have often succeeded in saving valuable teeth, that would have been considered hopeless cases a few years ago. But it is not to be denied, that a morbid action is sometimes kept up in the investing and alveolar membranes, to a greater or less extent; and, in some instances, the extraction of the teeth becomes absolutely necessary. I think this is seldom the case, however, unless the nerve cavity has been exposed for some time, and some degree of inflammation has extended to the periosteum previous to the operator.

I come now to speak of what many consider the only effectual remedy for tooth-ache, viz.

*Extracting Teeth.*—In many cases, it must be acknowledged, this is not only the most effectual remedy, but is rendered absolutely necessary, to put a stop to those morbid influences arising from diseased teeth, which are often of a very serious character.

Almost all, from the infant in the cradle to the man of old age, are obliged, at some period, to submit to this operation, or suffer the most serious consequences for neglecting it. And when we reflect upon its frequent necessity, together with the really serious difficulties sometimes to be encountered, and the advantages to be derived from it, so far as it regards its physical influences, it must be admitted that there is not an operation in surgery, that has higher claims upon the enlightened and humane operator. Every one who has had the least experience in this department of surgery, is aware that the difficulties to be encountered in the extraction of some teeth, is not worthy to be named in comparison with those that occur in the extraction of others; and that these difficulties will often occur unexpectedly. Hence, I insist, that this branch of our art has not received that attention which its importance demands. Every individual, who has ever had a truly aching molar, and has been obliged to submit to its extraction, need not be told, that there is a reality in the suffering that attaches itself to scarcely no other. The agony of an aching tooth, that is almost past endurance, added to the horror that seizes upon the minds of some, at the bare thought of extraction, are sufficient to convince every liberal minded man, that his reputation for skill in this department of surgery must be most unquestionable, in order to induce them to submit to an operation which is absolutely necessary. It is on occasions like these that the well-bred gentleman, and the scientific and skilful operator, will demean himself in such a manner as to secure the confidence and esteem of the suffering individual. His whole demeanor should show, that he has a heart to feel, a mind to plan, and a firm and skilful hand, which is capable of overcoming every obstacle, and of bringing the most difficult operation to a successful termination. If the operation is safely and speedily terminated, he will, usually, receive the hearty thanks of the person he has relieved.

I cannot forbear noticing here, a case that occurred in my practice in October, 1843. The subject was the daughter of captain F., about thirty-five years of age. Her father had, for many years, followed the seas, which had furnished him with all the comforts, and many of the luxuries that this world affords.

But that scourge of our race, phthisis pulmonalis, had been making inroads upon her vitals for many months. In her enfeebled state, she had, for a long time, been suffering pain in the left upper dens sapientiæ. She had resided much of her time in one of our large cities, though then residing in a most retired situation in the country, and had always availed herself of the skill of the first operators that our country could afford. Her physician being sensible that the morbid action, arising from the diseased tooth, was not only the cause of much suffering, but was exerting an unfavorable influence on her disease, advised its extraction. As the tooth was much decayed and difficult of access, and he had no instrument to extract a tooth of that description, except the turnkey, he advised her to send for me. Owing to some detention, and having several miles to travel, I did not reach there until evening, which I much regretted. Notwithstanding the kind reception with which I was met by the family, I found the lady, although she had suffered much misery from the tooth, for a long time, quite averse to having it extracted, and quite dubious, apparently, whether it could be effected. By kindness, and much persuasion, I finally induced her to permit me to look at the tooth, I found a large part of the crown decayed away, but by a thorough exploration, I satisfied myself, that by passing the beaks of the forceps as high up as possible upon the neck of the tooth, that I could remove it without serious difficulty. I immediately assured her that she might banish all her fears, that if she would simply extend her mouth, I would soon give her an opportunity of an occular examination of the cause of all her pain. With the aid of her friends, who had not, till this moment, heartily joined me, she was soon persuaded to submit to the operation. Though an exceedingly feeble delicate lady, she sat down with a degree of fortitude that was truly admirable, and, in a few seconds her tooth was in her hand. None but those who have witnessed a similar scene, can conceive the joy and gratitude that were manifested by the lady and the whole family.

I parted from the family, happy in being able to afford relief and comfort to one so amiable, and to enjoy their confidence and gratitude. I have recently learned, with regret, that this estimable lady is no more.

But to return. It is incumbent on the operator, to be able to extract a tooth or root in all cases where it is indicated, and with the least possible pain to the patient. In order to effect this, he will, I think, find the ordinary instruments, the turnkey, and, perhaps, one or two pairs of forceps, quite inadequate. If he has a good supply of forceps, well adapted to different classes of teeth, he will be able, advantageously, to dispense with the turnkey, probably, in ninety-nine cases out of one hundred. For the extraction of the lower molares, a well constructed turnkey may, sometimes, be advantageously used. There has been a vast improvement in the construction of these instruments, within a few years past, though perfection has not yet been attained.

A great desideratum, is to have the beaks of the instruments adapted to the precise shape of the teeth they are intended to extract. Many of the old forceps would seem to have been made, rather to cut off teeth than to extract them; and there is still a deficiency in almost all the instruments that are in use in the above particular. I have found the only way to obviate this difficulty, is to stand by the manufacturer, with the different classes of teeth at hand, to try the forceps upon them, and have them altered till they precisely fit. The most common fault is, that the extreme points of the beaks, press too firmly; the consequence is, that when pressure is made, the tooth is not firmly embraced, except at the extreme points of the instrument; is with much more difficulty disengaged from its connection in the socket, and is more liable to be crushed. The handles should also be adapted to the shape of the hand, with as much precision as the celebrated tailor's shears of Mr. Heinisch. Let us refer for a moment, to one or two points in the anatomical structure of the jaws. It will be found by observing the jaws in different subjects, that the external portion of the alveoli is the thinnest and most elastic, and that the upper jaw is more spongy than the lower.

I shall say nothing of the formation, developement and peculiarities of the different classes of teeth, both infant and adult; but would premise, that a minute knowledge on these subjects, will be found highly necessary to one who practices much upon the dental organism.

The operator having provided himself with a suitable supply of forceps, (and a score of them will not be found amiss,) should next furnish himself with a suitable gum-lancet. The blade should be narrow and thin, so that it will pass readily on the lateral surfaces between the teeth as well as on the exterior and interior, and should be passed down quite to the alveolus at every point. By this process the fabled ligamentum dentis will surely be severed. It has been proposed by some operators, recently, to dispense with this preliminary operation altogether; saying, that it only caused unnecessary pain to the patient, and that the beaks of the forceps would effect this object as well, and more expeditiously. But this, to my mind, appears to be a very harsh and unsurgical method of cutting the flesh, or detaching the gums from the tooth, and, farther, according to my observation, the strongest attachment is on the lateral surfaces, that face each other, and this part must remain unsevered, unless the lancet is resorted to. For the convenience of the operator, the patient should be placed on a high seat, with a support for the head, for the extraction of the upper teeth, and on a low one, for the extraction of the lower. A tumbler of water (tepid if the weather is cold) should be placed within reach of the patient. In this may be infused a little Cologne water, spts. camphor, or tinct. myrrh. After separating the gums, and rinsing the blood from the mouth, the operator should take time, thoroughly to explore the tooth and contiguous parts, and determine, as nearly as possible, what obstacles are to be met and overcome; though the experienced operator will usually do this at a glance. The patient should be seriously enjoined not to raise his hands during the operation, and this object will be more likely to be attained if he is directed to grasp, with a firm hand, the arms or rungs of the chair on which he sits.

The operator will next select the pair of forceps adapted to the case. A firm footing, and a firm and steady hand, are now indispensable. Let the head of the patient be firmly secured by the left-hand, while with the right, the beaks of the instrument, are cautiously, but expeditiously, adapted to the neck of the tooth. The instrument should now be passed up firmly to the alveolus, and almost at the same instant, it should receive a powerful traction, downward, and outward, if in the superior, and upward, and inward, if in the inferior jaw. This direction applies more particularly to the molars. Unless the attachment is stronger than ordinary, a change in the direction of the instrument from external to internal, and vice versa, will not be necessary. Should the attachment be unusually strong, a lateral motion should be effected from the external toward the internal part of the mouth, and should the beaks of the instrument slip in the least upon the tooth, or should there be danger of the tooth being crushed, they should be re-applied, and higher up if possible. In some cases, it is, undoubtedly, necessary to take off, with the beaks of the forceps, the points of the alveolus. This is particularly the case where the alveolus extends down low upon the neck of the tooth, where the attachment is exceedingly strong, and where the crown is decayed quite down to the alveolus.

When a molar tooth is thus situated, with irregular, crooked and spreading roots, the obstacles to be overcome are sometimes so great, that the roots cannot be extracted until a separation is made at the crown with a pair of splitting forceps. After a separation is effected, the roots that before defied every effort to lift them from their sockets, may be extracted with ease.

For the removal of the roots of molar teeth, forceps with slender beaks, curved at an angle of about forty-five degrees, should be used. The reasons why the instrument should deviate from the perpendicular, when traction is made, are obvious. If it does not, every fibrous attachment of the periosteum, together with the nerve cords and blood-vessels, must be severed at the same instant. This would require a muscular force that few possess; and could it be effected, the pain would be so intense as scarcely to be endured. Hence the elevators, and all instruments that were constructed to act on this principle, have signally failed. In extracting the superior molares, the inner root will be found to diverge more from the perpendicular than the two external, and this forms an additional reason for turning them outward.

It is often noticed, that the investing and alveolar membranes of dead roots, are in a state of ulceration, and the pus that is accumulated, makes its way through the alveoli and gums, in the form of gumboils. The morbid action that is kept up by roots in this condition, is much more injurious than is commonly supposed. In illustration of this remark, if the alveoli of the dead subject are carefully examined, their condition will determine at once, whether the teeth were healthy or otherwise. If the roots had been dead and in an ulcerated condition for any length of time, the alveoli will be found to be eroded and spongy like a honeycomb, and sometimes almost destroyed.

For the removal of roots that are decayed, even far below the alveolar processes, so that no ordinary instrument can reach them, I have found the conical screw, an instrument of great value; particularly for those of the incisors and cuspidati. In some cases of this kind, I know not how it can be dispensed with.

Though I have occupied far more of your time than I intended, I am still aware that I have not discussed the subject in a manner commensurate with its importance, but hope some useful ideas may have been suggested, and that more able and practical men, may give us the results of their experience, based on correct principles, which may serve to illuminate the pathway of future operators.

